# COVID-19 Vaccine Intention and Knowledge, Literacy, and Health Beliefs among Japanese University Students

**DOI:** 10.3390/vaccines10060893

**Published:** 2022-06-02

**Authors:** Takashi Miyachi, Yuta Sugano, Shizune Tanaka, Junko Hirayama, Fumio Yamamoto, Kyoko Nomura

**Affiliations:** 1 School of Medicine, Akita University, 1-1-1 Hondo, Akita 010-8543, Japan; takashi.miyachi02@gmail.com (T.M.); yutayuta20010829@gmail.com (Y.S.); snowdrop122@outlook.jp (S.T.); 2 Department of Environment Health Science and Public Health, Akita University Graduate School of Medicine, 1-1-1 Hondo, Akita 010-8543, Japan; junhirayama@med.akita-u.ac.jp; 3 Akita University, 1-1-1 Hondo, Akita 010-8543, Japan; f-yama@cvs.med.akita-u.ac.jp

**Keywords:** COVID-19 vaccine, vaccine hesitancy, vaccine acceptance, health belief model, university students

## Abstract

This study investigated the intention to get the coronavirus disease of 2019 (COVID-19) vaccine and its associated factors among Japanese university students. A cross-sectional survey was conducted from March to May 2021 via an e-learning platform at Akita University. Participants were 1776 graduate and undergraduate students who answered the survey on vaccine intention, the health belief model (HBM), sociodemographic characteristics, and concerns over COVID-19-related situations. Vaccine intention was stratified into active, slightly less, and no intention, and the associated factors were determined using the multinomial logistic regression model. Results showed that 56.7% of students had active intention, followed by slightly less intention (34.5%) and no intention (8.8%). After adjusting for covariates, healthcare course, perceived severity (life-threatening and serious social consequences), and perceived benefits from HBM were significantly associated with active intention, with adjusted odds ratios of 4.02 (95% confidence interval [CI], 2.11–7.67), 1.40 (95% CI, 1.16–1.69), 1.23 (95% CI, 1.04–1.46), and 2.03 (95% CI, 1.66–2.49), respectively; perceived barriers (side effect, troublesome, and parent disagreement) were adversely associated with active intention. The public health strategy to improve students’ vaccine uptake requires providing accurate information on vaccine safety and efficacy while removing any barriers to vaccination.

## 1. Introduction

The coronavirus disease of 2019 (COVID-19) poses a threat to public health, although this threat is gradually being tempered as vaccination coverage increases. Most countries have prioritized vaccination for high-risk populations, such as older people, those with chronic health conditions, and front-line health workers. Full vaccination significantly reduces the likelihood of infection and progression of the severe stage of pneumonia [1]. Consequently, many infection cases have been confirmed in unvaccinated populations, especially among young people. For example, in Japan, more than half of the 73,132 people with COVID-19 infection reported from 28 July to 3 August 2021, were aged 20–30 years, and they were mostly unvaccinated at that time [2]. Although newly mutated viruses are reported to infect even the vaccinated population [3], vaccines are still effective in preventing infection severity; therefore, increasing vaccination coverage remains a top agenda of public health policy [4].

People’s intention to be vaccinated is key to controlling the COVID-19 pandemic. Previous findings have suggested that young people comprise the group with the least interest in being vaccinated [5,6,7]. Since young people are less likely to become seriously ill even if they are infected by COVID-19, they may be less motivated to become vaccinated because of the risk of lethal side effects [8]. Concerns over the vaccine’s side effects are generally high since the COVID-19 vaccines were developed in a dramatically short period (compared with any other vaccine) through the application of new biotechnologies such as mRNA. Furthermore, young people rarely visit hospitals on a regular basis; thus, they have limited relationships with medical professionals whose reliable information encourages vaccine uptake [9,10,11,12], leaving the young people vulnerable to misinformation [13]. Moreover, the literature tends to exaggerate concerns about vaccines, which may exceed the perceived benefits, making young readers more reluctant to take vaccines [14,15,16].

Furthermore, the effectiveness of COVID-19 vaccines decreases over time as the antibody titer decreases; thus, specialists as well as pharmaceutical companies such as Pfizer recommend the implementation of booster shots [3]. In Japan, preparations for the third dose of COVID-19 vaccine were underway as of December 2021. However, given that young people are less likely to get their second doses (around 75%) compared to people in their 50s (around 90%) [17], it is important to identify the factors associated with vaccine intention among young people to develop timely public health strategies to increase the overall vaccine coverage in Japan. Therefore, this study aimed to reveal the COVID-19 vaccine intention and its associated characteristics among Japanese university students.

## 2. Methods

### 2.1. Study Participants and Survey Design

This cross-sectional study was part of the Student Mental Health Survey conducted at Akita University from 1 March to 31 May 2021. Akita University is one of the 47 national universities in Japan and has the largest student body in the Prefecture. Akita Prefecture has had the highest suicide rate among the 47 prefectures for the past 20 years and the mental health of young people, especially students, is an important issue. All students enrolled in the university (4370 undergraduates and 725 graduates) were approached via institutional emails and asked to log in to the e-classroom platform, where a link to the online self-administered questionnaire was prepared. On the first page of the online self-administered questionnaire, the purpose of the study was explained to the participants, and they were informed that their entire participation was voluntary and that the data collected in the study was confidential. They were also assured of their right to withdraw from the study at any time by submitting an opt-out withdrawal form without any repercussions regarding their academic records.

Details of the study methods and participant recruitment are described in our previous study [18]. Among 5111 students recruited, a total of 1982 completed the online self-administered questionnaire (response rate: 38.9%). After excluding participants who did not respond with their vaccine intention (*n* = 206), 1776 students became subjects for analyses. 

This study was approved by the Institutional Review Board of the Medical Ethical Committee at Akita University (No. 2520). 

### 2.2. Questionnaire and Measures 

#### 2.2.1. Outcome

The outcome for this study is university students’ intention to receive COVID-19 vaccine. Thus, the study participants were asked the question, “At what time do you intend to receive the COVID-19 vaccine?” They answered based on a six-point scale: “as soon as possible” = 1, “within six months” = 2, “within a year” = 3, “within three years” = 4, “no intention to receive a vaccine” = 5, and “not sure” = 6. Subsequently, the respondents were categorized based on ternary variables into the “active intention group” (response patterns 1 to 3), “slightly less intention group” (response patterns 4 and 6), and “no intention group” (response pattern 5).

#### 2.2.2. Health Belief Model (HBM)

HBM is a very widely utilized theory to predict preventive health-related behavior [19]. We utilized the HBM to understand what determines COVID-19 vaccine intention referring to previous studies investigating the association between the components of HBM and COVID-19 vaccine uptake and intention [20,21,22]. For the HBM, there was no specific scale, and an item question of the HBM used varies according to a study. In this study, before we determined the question items, we had performed an extensive search for health beliefs associated with vaccine intention among adolescents. The major components of the HBM included perception of (1) being susceptible to COVID infection and (2) being severe once infected, and (3) benefits of the vaccine, (4) barriers of vaccine uptake, and (5) cues to action. Among these, we identified that (1), (2), and (3) are mutually inclusive in previous literature, while (4) barriers and (5) cues to action are greatly modified according to real situations of the target infection. For example, “cues to action” related to COVID-19 infection refer to perceptions that health authorities recommend the vaccination or that a participant had those who died or suffered from COVID-19 among their relatives or acquaintances. In Japan, it was very clear that the government and health authorities highly recommend getting the vaccine, especially for young generations. In addition, at the time of our study investigation, the number of deaths had been few in Japan and thus a question item related to “cues to action” was not included in our study. For this reason, applying the original concept of the HBM, we included particular barriers such as the number of shots, feeling troublesome (reluctance), and parents’ opposition referring to the real situation under the COVID-19 pandemic in Japan. Accordingly, the components of HBM included in our study were perceived susceptibility, severity, benefits and barriers. Perceived susceptibility had two items: (1) I might get infected with COVID-19 (get an infection), and (2) I might spread COVID-19 if I get infected (spread infection). Perceived severity also had two items: (1) I believe that COVID-19 is a life-threating disease for me (life-threatening) and (2) I believe that there will be serious social consequences if I get infected (serious social consequences). Furthermore, perceived benefits had one item: I believe that a COVID-19 vaccine will protect me from getting infected. Perceived barriers had three items: (1) I am worried about the side effects of COVID-19 vaccine (side effect), (2) I feel that two shots of the vaccine are troublesome for me (troublesome), and (3) My parents do not agree with my taking the COVID-19 vaccine (parent disagreement). All components were measured based on a five-point Likert scale (1 = “agree” to 5 = “disagree”), and the scores were inverted so that higher scores corresponded to stronger health beliefs.

#### 2.2.3. Covariates

The other items investigated in this study were sex, age, study course, smoking habits, alcohol consumption (normal drinker: ethanol consumption < 140 g/week vs. heavy drinker: ethanol consumption ≥ 140 g/week), daily exercise, and concerns over the COVID-19 surrounding situations. Daily exercise habits were represented by metabolic equivalents (METs) * hours/week, after multiplying METs with the hours spent on daily exercise, frequency of weekly exercise, and values reflecting the intensity of exercise (light = 4, moderate = 6, vigorous = 8, and very vigorous = 10). Concerns over the COVID-19 surrounding situation, including those related to financial strain, academic career, leisure, social support, and physical activity since the outbreak of COVID-19, were assessed based on a 10-point Likert scale (1 = “not worried at all” to 10 = “extremely worried”). Consequently, the total score of concerns (Cronbach’s α = 0.704) was generated by adding the scores of the above five questions.

### 2.3. Statistical Analyses

First, we compared variables among the three vaccine intention groups (active intention vs. slightly less intention vs. no intention) and statistically assessed them using a one-way analysis of variance for continuous covariates or a chi-square test for categorical covariates. Second, we conducted a univariate multinomial logistic regression analysis to examine the variables’ association among the three groups. Third, adjusting for all covariates in the models, a multinomial logistic regression analysis was carried out to estimate odds ratios (ORs) along with 95% confidence intervals (CIs). The no intention group was set as the reference group. Internal consistency of concerns over the COVID-19 surrounding situation was assessed using the Cronbach’s α coefficient, and it was adapted as the total score of concerns in the logistic regression analysis. 

All analyses were performed using SAS software (version 9.4; SAS Institute, Cary, NC, USA). The statistical significance value was set as the two-sided *p*-value < 0.05.

## 3. Results

### 3.1. Sociodemographic Background, HBM, and Concerns over the COVID-19 Sorrounding Situation According to Vaccine Intention

As Table 1 shows, a total of 1776 students (mean age, 20.9 ± 4.1 years) participated in the survey: 53.6% were male and one-fourth belonged to the healthcare course. More than half of the students (*n* = 1007, 56.7%) had active vaccine intention; this was followed by the slightly less intentional group (*n* = 613, 34.5%) and the no intention group (*n* = 156, 8.8%). Students in the active intention group were associated with the healthcare course (*p* < 0.001), whereas those in the no intention group were more likely to smoke (*p* = 0.062) and drink heavy amounts of alcohol (*p* < 0.056).

Regarding the HBM components, students in the active intention group were more likely to have higher scores on the two items of perceived susceptibility (get an infection [*p* < 0.001] and spread infection [*p* = 0.006]), two items of perceived severity (life-threating [*p* < 0.001] and serious social consequences [*p* < 0.001]), and perceived benefit (*p* < 0.001). Furthermore, students in the no intention group were more likely to have higher scores on the three items of perceived barriers (side effect [*p* < 0.001], troublesome [*p* < 0.001], and parent disagreement [*p* < 0.001]). For concerns over the COVID-19 surrounding situation, students in the no intention group tended to score higher on all items (*p* < 0.01).

### 3.2. Multinomial Logistic Regression Analyses of Vaccine Intention 

**Figure 1:**
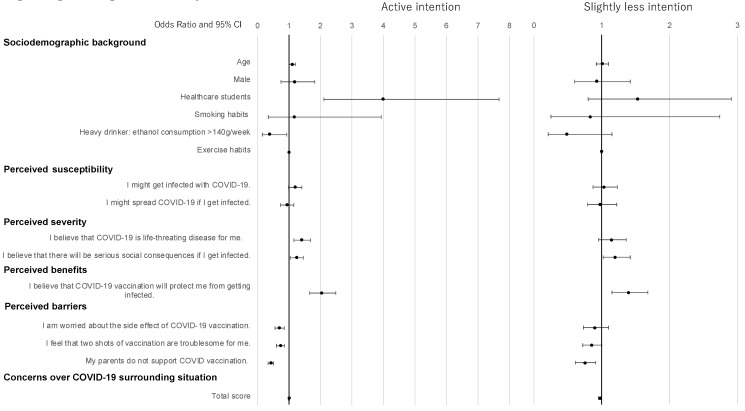
Logistic regression analyses of vaccine intention.

Table 2 and Figure 1 show the results of univariate and multinomial logistic regression analyses according to vaccine intention. Adjusting for age, sex, study course, smoking, drinking and exercise habits, and total scores of concerns, the multinomial models demonstrated that one unit of age (OR 1.10, 95% CI, 1.01–1.21) and students in the healthcare course (OR, 4.02, 95% CI, 2.11–7.67) compared to those in other courses were significantly associated with active intention. For perceived severity, life-threatening perception (OR, 1.40, 95% CI, 1.16–1.69) was significantly associated with an increased risk of active intention compared to no intention. The perception of serious social consequences was significantly associated with an increased risk of both active (OR, 1.23, 95% CI, 1.04–1.46) and slightly less intention (OR, 1.20, 95% CI, 1.02–1.42) compared to no intention. Perceived benefits had a 2.03-fold increased risk of active intention (95% CI, 1.66–2.49) and 1.39-fold increased risk of slightly less intention (95% CI, 1.15–1.68) compared to no intention. For perceived barriers, one unit increase of side effect and feeling troublesome were associated with a decreased risk of active intention, with the adjusted ORs of 0.69 (95% CI, 0.56–0.85) and 0.73 (95% CI, 0.61–0.86), respectively. For the barrier of parent disagreement, active and slightly less intention were lineary and inversely associated with one unit increase of parent disagreement compared to no intention, with the adjusted ORs of 0.42 (95% CI, 0.34–0.51) and 0.75 (95% CI, 0.61–0.91), respectively. Similarly, one unit increase of the total scores of concerns was inversely associated with slightly less intention compared to no intention, but the association was not significant with regard to active intention. 

## 4. Discussion

We investigated the COVID-19 vaccine intention among students at a Japanese university and found that 56.7% of the study participants had an active vaccine intention. In contrast, 34.5% had slightly less intention, and the remaining 8.8% had no intention. We used HBM to identify the factors associated with their intention and generally confirmed that perceived severity (life-threatening and serious social consequences), and perceived benefits were associated with vaccine intention, whereas perceived barriers (side effects, troublesome and parent disagreement) were inversely associated with such intention. Moreover, there was a clear difference in vaccine intention between students belonging to the healthcare course and other courses, with those in the healthcare course having a substantially higher percentage of active intention. Here, we discuss our findings in light of the previous literature and study limitations. 

Among the total of 1776 student participants, the active intention group accounted for 56.7%. This survey was conducted between March and May of 2021, when COVID-19 vaccination in Japan was limited to healthcare workers and older people. Our findings are consistent with previous reports on lower vaccine intention among young people compared with other age groups under similar circumstances. For example, a survey conducted in Japan in February 2021, before vaccination of older people began, indicated that 64.2% of them intended to be vaccinated. In the same survey, the vaccine acceptance rate of people in their 20s was only 37.7% [23]. Although our subjects showed slightly higher vaccine acceptance rates, it is worth noting that, as of January 2021, before the vaccination for healthcare workers began, the percentage of Japanese people aged 20–49 years with vaccine intention was 63.6%. These trends indicate that the vaccine intention of young people has not changed much since the begining of 2021 [23]. In contrast, 8.8% of respondents did not have vaccine intention in our study, which is slightly lower than the 9.8% for Japanese people in the same age group who were unwilling to receive the vaccine in the February 2021 survey [24]. These results are consistent with the latest systematic review of COVID-19 vaccination preferences, which found that around 14% of participants expressed opposition to vaccination [5]. 

The percentage of the active intention group among our participants was quite a bit lower than that of university students in other countries. While the numbers varied depending on the research method and timing, surveys conducted before the vaccine campaign for young people began indicated that the percentage of young people likely to become vaccinated was 79.6% in Canadian universities [25], 86.1% in Italy [26], and 91.6% in the United States [27]. Such high vaccine intention in Western countries could be attributed to more stringent preventive measures, with less success of control over the COVID-19 pandemic [28]. In contrast, low percentage of active intention was also observed among Chinese university students (76.3%) [29]. However, low acceptability (i.e., low active intention) of getting a COVID-19 vaccine in our study may be greatly influenced by a historical event associated with the human papilloma virus (HPV) vaccine [30]. In Japan, when the HPV vaccine was introduced in the routine vaccination package in 2013, many young girls suffered from unknown neurological symptoms associated with the vaccine. Some of them sued the Ministry of Health, Labour, and Welfare, and the Japanese government stopped the active promotion of the HPV vaccine just two months after its introduction [31]. Although active vaccine promotion resumed in December 2021 [32], young girls who should have been vaccinated were not vaccinated for eight years, which could have an alarming impact on the incidence of cervical cancer in Japan [33]. With such a significant impact of the vaccine-related adverse reaction, Japanese people may be more cautious about vaccines, especially foreign-made vaccines. A systematic review identified parental attitudes and concerns as barriers to administering the HPV vaccine to adolescents [34]. Thus, continued efforts are needed to ensure that both parents and young people understand the importance of active vaccine promotion.

In our study, among the components of HBM, perceived severity and perceived benefit were significantly associated with vaccine intention, while perceived barriers were inversely associated with intention. In perceived severity, serious social consequences were associated with both active and less intention compared to no intention, while life-threatening perception was only associated with active intention. These findings may suggest that the perception of serious social consequences rather than life-threatening perception was consistently associated with vaccine intention. Indeed, the total numbers of COVID-19 infection cases and deaths in Japan are still low compared with those in Western countries. Therefore, young people in Japan tend to take COVID-19 less seriously because the initial variant of the virus was not highly lethal for them [8], but they are rather concerned about the potential social impact on their life if they become infected. In Japan, the stigma associated with COVID-19 that flourished with dramatic stories in the media and through the internet sometimes results in discrimination against the infected groups, which caused negative consequences both for the individuals and for society. Previous studies also identified that university students who believed that COVID-19 was not a severe disease were less likely to take the vaccine [22,35]. Considering the unknown threats posed by the new mutant strains [36] and the possible long-term consequences of the infection, such as a taste disorder [37], it is necessary to inform young people of the comprehensive effects of COVID-19 in addition to its severity. 

We also found that the perceived benefit of vaccine protection was linearly and significantly associated with active and slightly less intention. The impact of the perceived benefits on vaccination behavior among college students and the general population has also been reported [26,38]. Although the current vaccine does not fully prevent COVID-19 infection, it has been shown to be effective in preventing severe disease symptoms [3,4]. Since the public does not necessarily know about the latest evidence regarding COVID-19 and the vaccines, communication strategies to deliver appropriate information to the public is important [39]. There have been many reports that concerns about vaccination side effects hinder its uptake [12,40,41]. Mant et al. [25] found that 80% of students who were unwilling to take the vaccine were worried about the exceptional speed of the vaccine’s development, suggesting the need for high transparency about the vaccine’s research and development process. In our study, parental disagreemet was linearly and significantly associated with both active and slightly less intention compared to no intention. Parental influence on students’ decision-making regarding vaccination has also been reported in previous research [42]. In this regard, the linear result of parental opposition with active and slightly less intention in our study underscored the importance of parental understanding of the impact of COVID-19 on health, and the efficacy and safety of the COVID-19 vaccine. 

Furthermore, we found a significantly higher percentage of active intention among students in a healthcare course compared with students in other courses, while slightly less or no intention was significantly higher among students in other courses. Riad et al. [43] reported that, in the Czech Republic, a significantly higher percentage of healthcare students were willing to be vaccinated than non-healthcare students (80% vs. 68.7%), whereas non-healthcare students were more likely to be unwilling (22.6%) or unsure (14.6%) regarding vaccination compared with healthcare students (8.7% and 5.5%, respectively). This remarkable difference between the students can be attributed to the communities to which they belong. Healthcare students tend to have access to accurate information about vaccines because they are in close proximity with medical professionals and students undergoing hospital training. In addition, most healthcare professionals were already vaccinated at the time of this investigation, which might have motivated the students to get vaccinated. Indeed, previous studies have shown that recommendations by surrounding medical professionals and being around people who are vaccinated are important factors promoting vaccination [12,22,24,43]. 

Some studies underscored the importance of students’ concerns regarding their own health and well-being [44]. The association between students’ concerns about their general life and vaccine acceptance are inconsistent among different countries since the students are influenced by infection statistics and policy (i.e., number of deaths, collapsed medical care system, vaccine passport discussion, etc.). Our study revealed that students’ concerns, particularly about leisure, social support, and physical activities, were significantly higher among the no vaccine intention group. Of note, more than 90% of this group’s participants belonged to non-healthcare courses. This suggests that stringent infection control measures such as campus closures, restriction of club activities, and restriction on travel outside the prefecture might have deprived these students of the opportunity to interact with each other and to be part of society, resulting in their social isolation [45]. Under such circumstances, non-healthcare students were more likely to be exposed to misinformation circulated on social media, have less opportunity to obtain accurate information from health professionals, have lower health knowledge, and be at greater risk of believing in conspiracy theories than students in the healthcare course [46,47]. Thus, as we are now confronted with the new public health agenda of a vaccine booster dose, misinformation about vaccination could easily spread because of students who are reluctant about vaccination. Universities and societies are required to analyze data about students’ intention and the surrounding associated factors while making continuous efforts to provide them with accurate information on vaccine safety, effectiveness, and limitations.

Despite the strengths of this study, it has several limitations that need to be addressed. First, since the study was conducted on students at a single university in Japan, the findings’ generalizability might be limited. However, Akita University is one of 47 national universities in the 47 prefectures of Japan, and it has the largest student body in the Prefecture. Our result could be generalized to similar universities in other prefectures. Second, this study investigated vaccine intention and might not reflect the actual vaccine behavior. Since we did not measure actual vaccine uptake, we were unable to verify the relationship. Third, vaccination is done on a voluntary basis in Japan, but there is invisible pressure on all people in the society to get vaccinated. This study was conducted by the university where the students studied. Therefore, more students might have shown a pro-vaccine attitude against their will due to the social-desirability bias. Finally, the cross-sectional design did not allow us to make causal inferences about the relationships among the variables.

## 5. Conclusions

We investigated the COVID-19 vaccine intention and its associated factors among Japanese university students. We found that only half of the students had active intention, whereas the other half had slightly less or no intention. The public health strategy to improve vaccine uptake among university students requires continued efforts to provide accurate information on morbidity (severity) of disease consequences and the vaccines’ efficacy (prevention) to vaccine recipients, in addition to providing information on vaccine safety to their parents.

## Figures and Tables

**Table 1:** Sociodemographic background, health belief model (HBM), and concerns over the COVID-19 surrounding situation according to vaccine intention.

		Total		Vaccine Intention						*p*-Value ^c^
				Active Intention		Slightly Less Intention		No Intention		
		(N = 1776)		(N = 1007; 56.7%)		(N = 613; 34.5%)		(N = 156; 8.8%)		
**Sociodemographic background**										
Age	Years ^a^	20.9 ± 4.1		21.3 ± 4.8		20.2 ± 3.0		20.3 ± 1.9		<0.001
Sex	Female	824	(46.4)	461	(45.8)	300	(49.0)	63	(40.4)	0.128
	Male	951	(53.6)	546	(54.2)	312	(51.0)	93	(59.6)	
Study course	Non-healthcare	1323	(74.6)	667	(66.4)	515	(84.2)	141	(90.4)	<0.001
	Healthcare	450	(25.4)	338	(33.6)	97	(15.9)	15	(9.6)	
Smoking habits	No	1721	(97.3)	974	(97.1)	600	(98.2)	147	(94.8)	0.062
	Yes	48	(2.7)	29	(2.9)	11	(1.8)	8	(5.2)	
Drinking habits	g/week ^a^	20.7 ± 58.7		22.5 ± 61.3		15.5 ± 46.9		29.6 ± 79.2		0.010
	Normal (ethanol consumption < 140 g/week)	1664	(95.5)	944	(95.5)	582	(96.5)	138	(92.0)	0.056
	Heavy (ethanol consumption ≥ 140 g/week)	78	(4.5)	45	(4.6)	21	(3.5)	12	(8.0)	
Exercise habits	METs * hour/week	21.2 ± 44.8		22.7 ± 54.6		18.5 ± 25.6		21.7 ± 31.0		0.197
**Components of the HBM ^a^**										
*Perceived susceptibility*										
I might get infected with COVID-19.		3.8 ± 1.2		4.0 ± 1.1		3.7 ± 1.2		3.5 ± 1.3		<0.001
I might spread COVID-19 if I get infected.		4.4 ± 0.9		4.5 ± 0.9		4.4 ± 0.9		4.2 ± 1.1		0.006
*Perceived severity*										
I believe that COVID-19 is a life-threating disease for me.		3.7 ± 1.2		3.8 ± 1.2		3.7 ± 1.2		3.3 ± 1.4		<0.001
I believe that there will be serious social consequences if I get infected.		3.8 ± 1.2		3.9 ± 1.2		3.8 ± 1.2		3.3 ± 1.4		<0.001
*Perceived benefits*										
I believe that a COVID-19 vaccine will protect me from getting infected.		3.7 ± 1.0		3.9 ± 0.9		3.5 ± 1.0		2.9 ± 1.2		<0.001
*Perceived barriers*										
I am worried about the side effects of COVID-19 vaccine.		3.7 ± 1.3		3.5 ± 1.3		4.0 ± 1.2		4.1 ± 1.3		<0.001
I feel that two shots of the vaccine are troublesome for me.		2.8 ± 1.3		2.6 ± 1.3		3.1 ± 1.3		3.5 ± 1.4		<0.001
My parents do not agree with my taking the COVID-19 vaccine.		2.3 ± 1.1		1.9 ± 1.1		2.7 ± 1.0		3.0 ± 1.2		<0.001
**Concerns over the COVID-19 surrounding situation (α = 0.704) ^b^**										
Financial strain		3.3 ± 2.6		3.3 ± 2.6		3.1 ± 2.5		3.9 ± 2.9		0.004
Academic career		3.6 ± 2.6		3.4 ± 2.5		3.6 ± 2.5		4.1 ± 2.9		0.003
Leisure		5.9 ± 3.1		6.1 ± 3.0		5.4 ± 3.1		6.2 ± 3.4		<0.001
Social support		4.4 ± 3.0		4.5 ± 3.0		4.1 ± 2.9		4.7 ± 3.2		0.007
Physical activity		3.7 ± 2.6		3.7 ± 2.6		3.4 ± 2.5		4.1 ± 2.7		0.003
Total score		20.8 ± 9.5		21.1 ± 9.4		19.6 ± 9.2		23.1 ± 10.6		<0.001

Notes: ^a^ The plus and minus values indicate means ± standard deviation. Numbers and percentages are shown for categorical variables. ^b^ The Cronbach’s α indicates internal consistency. ^c^ One-way analyses of variance for continuous variables or chi-square test for categorial variables. MET * = metabolic equivalent. HBM = Health Belief Model.

**Table 2:** Logistic regression analyses of vaccine intention.

		Vaccine Intention					
		Active Intention			Slightly Less Intention		
		Crude OR	*p*-Value	Adjusted OR (95% CI) ^a^	Crude OR	*p*-Value	Adjusted OR (95% CI) ^a^
**Sociodemographic background**							
Age	Years	1.10	0.008	1.10 (1.01–1.21)	1.00	0.897	1.01 (0.92–1.10)
Sex	Female	ref	0.208	ref	Ref	0.055	ref
	Male	0.80		1.17 (0.75–1.82)	0.71		0.92 (0.60–1.42)
Study course	Non-healthcare	ref	<0.001	ref	Ref	0.052	ref
	Healthcare	4.76		4.02 (2.11–7.67)	1.77		1.53 (0.80–2.91)
Smoking habits	No	ref	0.140	ref	Ref	0.022	ref
	Yes	0.55		1.16 (0.35–3.93)	0.34		0.83 (0.25–2.74)
Drinking habits	Normal (ethanol consumption < 140 g/week)	ref	0.075	ref	Ref	0.019	ref
	Heavy (ethanol consumption ≥ 140 g/week)	0.55		0.38 (0.16–0.93)	0.42		0.49 (0.21–1.15)
Exercise habits	METs * hour/week	1.00	0.816	1.00 (1.00–1.01)	1.00	0.222	1.00 (0.99–1.00)
**Components of the health belief model ^a^**							
*Perceived susceptibility*							
I might get infected with COVID-19.		1.41	<0.001	1.18 (0.99–1.41)	1.14	0.060	1.03 (0.87–1.23)
I might spread COVID-19 if I get infected.		1.29	0.002	0.93 (0.73–1.16)	1.19	0.043	0.98 (0.79–1.22)
*Perceived severity*							
I believe that COVID-19 is life-threating disease for me.		1.40	<0.001	1.40 (1.16–1.69)	1.26	0.001	1.14 (0.95–1.36)
I believe that there will be serious social consequences if I get infected.		1.44	<0.001	1.23 (1.04–1.46)	1.26	0.001	1.20 (1.02–1.42)
*Perceived benefits*							
I believe that a COVID-19 vaccine will protect me from getting infected.		2.61	<0.001	2.03 (1.66–2.49)	1.60	<0.001	1.39 (1.15–1.68)
*Perceived barriers*							
I am worried about the side effects of COVID-19 vaccine.		0.64	<0.001	0.69 (0.56–0.85)	0.88	0.126	0.90 (0.73–1.10)
I feel that two shots of the vaccine are troublesome for me.		0.56	<0.001	0.73 (0.61–0.86)	0.78	<0.001	0.85 (0.72–1.00)
My parents do not agree with my taking the COVID vaccine.		0.37	<0.001	0.42 (0.34–0.51)	0.72	<0.001	0.75 (0.61–0.91)
**Concerns over the COVID-19 surrounding situation (Cronbach’s α = 0.704)**							
Total score		0.98	0.015	1.00 (0.98–1.02)	0.96	<0.001	0.97 (0.95–0.99)

Notes: ^a^ Adjusted for variables with *p* < 0.25 in the univariate logistic analysis. OR = odds ratio, CI = confidence interval, MET * = metabolic equivalent.

## Data Availability

The data presented in this study are available on request from the corresponding author.

## References

[1] Mallapaty S. (2021). COVID vaccines slash viral spread—But Delta is an unknown. Nature.

[2] Ministry of Health, Labour and Welfare (2021). Visualizing the Data: Information on COVID-19 Infections. https://covid19.mhlw.go.jp/en/.

[3] Bergwerk M., Gonen T., Lustig Y., Amit S., Lipsitch M., Cohen C., Mandelboim M., Levin E.G., Rubin C., Indenbaum V. (2021). COVID-19 Breakthrough infections in vaccinated health care workers. N. Engl. J. Med..

[4] Thompson M.G., Burgess J.L., Naleway A.L., Tyner H., Yoon S.K., Meece J., Olsho L.E.W., Caban-Martinez A.J., Fowlkes A.L., Lutrick K. (2021). Prevention and attenuation of COVID-19 with the BNT162b2 and mRNA-1273 vaccines. N. Engl. J. Med..

[5] Robinson E., Jones A., Lesser I., Daly M. (2021). International estimates of intended uptake and refusal of COVID-19 vaccines: A rapid systematic review and meta-analysis of large nationally representative samples. Vaccine.

[6] Rosen B., Waitzberg R., Israeli A., Hartal M., Davidovitch N. (2021). Addressing vaccine hesitancy and access barriers to achieve persistent progress in Israel’s COVID-19 vaccination program. Isr. J. Health Policy Res..

[7] Baack B.N., Abad N., Yankey D., Kahn K.E., Razzaghi H., Brookmeyer K., Kolis J., Wilhelm E., Nguyen K.H., Singleton J.A. (2021). COVID-19 vaccination coverage and intent among adults aged 18–39 years—United States, March–May 2021. MMWR Morb. Mortal. Wkly. Rep..

[8] O’Driscoll M., Dos Santos G.R., Wang L., Cummings D.A.T., Azman A.S., Paireau J., Fontanet A., Cauchemez S., Salje H. (2021). Age-specific mortality and immunity patterns of SARS-CoV-2. Nature.

[9] Reiter P.L., Pennell M.L., Katz M.L. (2020). Acceptability of a COVID-19 vaccine among adults in the United States: How many people would get vaccinated?. Vaccine.

[10] Bish A., Yardley L., Nicoll A., Michie S. (2011). Factors associated with uptake of vaccination against pandemic influenza: A systematic review. Vaccine.

[11] Lau J.S., Adams S.H., Boscardin W.J., Irwin C.E. (2014). Young adults’ health care utilization and expenditures prior to the affordable care act. J. Adolesc. Health.

[12] Nomura S., Eguchi A., Yoneoka D., Kawashima T., Tanoue Y., Murakami M., Sakamoto H., Maruyama-Sakurai K., Gilmour S., Shi S. (2021). Reasons for being unsure or unwilling regarding intention to take COVID-19 vaccine among Japanese people: A large cross-sectional national survey. Lancet Reg. Health West. Pac..

[13] Roozenbeek J., Schneider C.R., Dryhurst S., Kerr J., Freeman A.L.J., Recchia G., van der Bles A.M., van der Linden S. (2020). Susceptibility to misinformation about COVID-19 around the world. R. Soc. Open Sci..

[14] Karlsson L.C., Soveri A., Lewandowsky S., Karlsson L., Karlsson H., Nolvi S., Karukivi M., Lindfelt M., Antfolk J. (2021). Fearing the disease or the vaccine: The case of COVID-19. Personal. Individ. Differ..

[15] Wong L.P., Alias H., Wong P.F., Lee H.Y., Abubakar S. (2020). The use of the health belief model to assess predictors of intent to receive the COVID-19 vaccine and willingness to pay. Hum. Vaccines Immunother..

[16] Zampetakis L.A., Melas C. (2021). The health belief model predicts vaccination intentions against COVID-19: A survey experiment approach. Appl. Psychol. Health Well-Being.

[17] Current C. 19 Situation and Vaccination Coverage. 65th Advisory Board Meeting of Ministry of Health, Labour, and Welfare, Japan. https://www.mhlw.go.jp/content/10900000/000875163.pdf.

[18] Nomura K., Minamizono S., Maeda E., Kim R., Iwata T., Hirayama J., Ono K., Fushimi M., Goto T., Mishima K. (2021). Cross-sectional survey of depressive symptoms and suicide-related ideation at a Japanese National University during the COVID-19 stay-home order. Environ. Health Prev. Med..

[19] Rosenstock I.M. (1974). The health belief model and preventive health behavior. Health Educ. Monogr..

[20] Chen F., He Y., Shi Y. (2022). Parents’ and guardians’ willingness to vaccinate their children against COVID-19: A systematic review and meta-analysis. Vaccines.

[21] Khalafalla H.E., Tumambeng M.Z., Halawi M.H.A., Masmali E.M.A., Tashari T.B.M., Arishi F.H.A., Shadad R.H.M., Alfaraj S.Z.A., Fathi S.M.A., Mahfouz M.S. (2022). COVID-19 vaccine hesitancy prevalence and predictors among the students of Jazan University, Saudi Arabia using the health belief model: A cross-sectional study. Vaccines.

[22] Patwary M.M., Bardhan M., Disha A.S., Hasan M., Haque M.Z., Sultana R., Hossain M.R., Browning M.H.E.M., Alam M.S., Sallam M. (2021). Determinants of COVID-19 vaccine acceptance among the adult population of Bangladesh using the health belief model and the theory of planned behavior model. Vaccines.

[23] Machida M., Nakamura I., Kojima T., Saito R., Nakaya T., Hanibuchi T., Takamiya T., Odagiri Y., Fukushima N., Kikuchi H. (2021). Acceptance of a COVID-19 vaccine in Japan during the COVID-19 pandemic. Vaccines.

[24] Kadoya Y., Watanapongvanich S., Yuktadatta P., Putthinun P., Lartey S.T., Khan M.S.R. (2021). Willing or hesitant? A socioeconomic study on the potential acceptance of COVID-19 vaccine in Japan. Int. J. Environ. Res. Public Health.

[25] Mant M., Aslemand A., Prine A., Holland A.J. (2021). University students’ perspectives, planned uptake, and hesitancy regarding the COVID-19 vaccine: A multi-methods study. PLoS ONE.

[26] Barello S., Nania T., Dellafiore F., Graffigna G., Caruso R. (2020). “Vaccine hesitancy” among university students in Italy during the COVID-19 pandemic. Eur. J. Epidemiol..

[27] Graupensperger S., Abdallah D.A., Lee C.M. (2021). Social norms and vaccine uptake: College students’ COVID vaccination intentions, attitudes, and estimated peer norms and comparisons with influenza vaccine. Vaccine.

[28] Han E., Tan M.M.J., Turk E., Sridhar D., Leung G.M., Shibuya K., Asgari N., Oh J., García-Basteiro A.L., Hanefeld J. (2020). Lessons learnt from easing COVID-19 restrictions: An analysis of countries and regions in Asia Pacific and Europe. Lancet.

[29] Bai W., Cai H., Liu S., Liu H., Qi H., Chen X., Liu R., Cheung T., Su Z., Ng C.H. (2021). Attitudes toward COVID-19 vaccines in Chinese college students. Int. J. Biol. Sci..

[30] de Figueiredo A., Simas C., Karafillakis E., Paterson P., Larson H.J. (2020). Mapping global trends in vaccine confidence and investigating barriers to vaccine uptake: A large-scale retrospective temporal modelling study. Lancet.

[31] Gilmour S., Kanda M., Kusumi E., Tanimoto T., Kami M., Shibuya K. (2013). HPV Vaccination programme in Japan. Lancet.

[32] JIJI Press Network of European World Shops (2021). Japan to Resume Active Promotion of HPV Vaccinations. https://sp.m.jiji.com/english/show/16079.

[33] Ikeda S., Ueda Y., Yagi A., Matsuzaki S., Kobayashi E., Kimura T., Miyagi E., Sekine M., Enomoto T., Kudoh K. (2019). HPV vaccination in Japan: What is happening in Japan?. Expert Rev. Vaccines.

[34] Holman D.M., Benard V., Roland K.B., Watson M., Liddon N., Stokley S. (2014). Barriers to human papillomavirus vaccination among US adolescents: A systematic review of the literature. JAMA Pediatr..

[35] Qiao S., Tam C.C., Li X. (2022). Risk exposures, risk perceptions, negative attitudes toward general vaccination, and COVID-19 vaccine acceptance among college students in South Carolina. Am. J. Health Promot..

[36] Twohig K.A., Nyberg T., Zaidi A., Thelwall S., Sinnathamby M.A., Aliabadi S., Seaman S.R., Harris R.J., Hope R., Lopez-Bernal J. (2022). Hospital admission and emergency care attendance risk for SARS-CoV-2 Delta (B.1.617.2) compared with Alpha (B.1.1.7) variants of concern: A cohort study. Lancet Infect. Dis..

[37] Nalbandian A., Sehgal K., Gupta A., Madhavan M.V., McGroder C., Stevens J.S., Cook J.R., Nordvig A.S., Shalev D., Sehrawat T.S. (2021). Post-acute COVID-19 syndrome. Nat. Med..

[38] Shmueli L. (2021). Predicting intention to receive COVID-19 vaccine among the general population using the health belief model and the theory of planned behavior model. BMC Public Health.

[39] Hyland-Wood B., Gardner J., Leask J., Ecker U.K.H. (2021). Toward effective government communication strategies in the era of COVID-19. Humanit. Soc. Sci. Commun..

[40] Machingaidze S., Wiysonge C.S. (2021). Understanding COVID-19 vaccine hesitancy. Nat. Med..

[41] Chido-Amajuoyi O.G., Talluri R., Shete S.S., Shete S. (2021). Safety concerns or adverse effects as the main reason for human papillomavirus vaccine refusal: National immunization survey—Teen, 2008 to 2019. JAMA Pediatr..

[42] Sandler K., Srivastava T., Fawole O.A., Fasano C., Feemster K.A. (2020). Understanding vaccine knowledge, attitudes, and decision-making through college student interviews. J. Am. Coll. Health.

[43] Riad A., Pokorná A., Antalová N., Krobot M., Zviadadze N., Serdiuk I., Koščík M., Klugar M. (2021). Prevalence and drivers of COVID-19 vaccine hesitancy among Czech university students: National cross-sectional study. Vaccines.

[44] Hawley S.R., Thrivikraman J.K., Noveck N., St. Romain T., Ludy M.-J., Barnhart L., Chee W.S.S., Cho M.J., Chong M.H.Z., Du C. (2021). Concerns of college students during the COVID-19 pandemic: Thematic perspectives from the United States, Asia, and Europe. J. Appl. Learn. Teach..

[45] Tahara M., Mashizume Y., Takahashi K. (2021). Mental health crisis and stress coping among healthcare college students momentarily displaced from their campus community because of COVID-19 restrictions in Japan. Int. J. Environ. Res. Public Health.

[46] Szmyd B., Bartoszek A., Karuga F.F., Staniecka K., Błaszczyk M., Radek M. (2021). Medical students and SARS-CoV-2 vaccination: Attitude and behaviors. Vaccines.

[47] Sallam M., Dababseh D., Yaseen A., Al-Haidar A., Ababneh N.A., Bakri F.G., Mahafzah A. (2020). Conspiracy beliefs are associated with lower knowledge and higher anxiety levels regarding COVID-19 among students at the University of Jordan. Int. J. Environ. Res. Public Health.

